# Isolation of a Trypanosome Related to* Trypanosoma theileri* (Kinetoplastea: Trypanosomatidae) from* Phlebotomus perfiliewi* (Diptera: Psychodidae)

**DOI:** 10.1155/2018/2597074

**Published:** 2018-07-15

**Authors:** Mattia Calzolari, Gianluca Rugna, Emanuela Clementi, Elena Carra, Marco Pinna, Federica Bergamini, Massimo Fabbi, Michele Dottori, Luciano Sacchi, Jan Votýpka

**Affiliations:** ^1^Istituto Zooprofilattico Sperimentale della Lombardia e dell'Emilia Romagna “B. Ubertini”, via Bianchi 9, 25124 Brescia, Italy; ^2^Dipartimento di Biologia e Biotecnologie “L. Spallanzani”, Università degli Studi di Pavia, via Ferrata 9, 27100 Pavia, Italy; ^3^Department of Parasitology, Faculty of Science, Charles University, Prague, Czech Republic

## Abstract

The* Trypanosoma theileri* group includes several trypanosome species hardly distinguishable due to the lack of discriminating morphological characters. Trypanosomes belonging to this group have been isolated from different bovine, ovine, and cervids in Europe, Africa, Asia, and Americas. The principal vectors of the* T. theileri* group are considered tabanid flies; however,* T. melophagium* is transmitted exclusively by sheep keds. In 2016, 128 sand flies out of 2,728 trapped in Valsamoggia municipality, Italy, were individually dissected and an unknown trypanosome strain, named TrPhp1, was isolated from a female of the sand fly* Phlebotomus perfiliewi*. Sequence analysis placed this trypanosome in the* T. theileri* group with very high homology to other trypanosomes detected in European cervids. This is the first report of the* T. theileri* group isolation from a sand fly, and the possible role of this insect group in the trypanosome transmission cycle is discussed. Within the* T. theileri* group, the phylogenetic analysis distinguished several lineages, which, unfortunately, do not correspond with their host specificity and their taxonomic status remains ambiguous.

## 1. Introduction

Genus* Trypanosoma* Gruby, 1843 (Euglenozoa: Kinetoplastea: Trypanosomatidae), infects all classes of vertebrate hosts, but most attention is directed to species that cause human and animal diseases and heavy economic losses [1]. Some trypanosomes can be highly pathogenic for their hosts including humans, such as the causative agents of Chagas disease (*T. cruzi*) or sleeping sickness (*T. brucei*); other species are considered to be veterinary important parasites causing serious diseases (e.g., nagana, surra, and dourine) and significant losses in livestock, mainly in Africa and Asia. In Europe, two trypanosome subgenera have been identified in ungulates. While* Trypanosoma evansi* and* T. equiperdum* (subgenus* Trypanozoon*) may result in fatal diseases of their hosts, members of the subgenus* Megatrypanum* (*Trypanosoma theileri*,* T. cervi*,* T. melophagium*,* T. stefanskii*, and* T. theodori* and other species described in the past on morphological basis) are generally considered to be nonpathogenic.

Within the subgenus* Megatrypanum*, the* Trypanosoma theileri* group includes trypanosomes of at least three well-defined species with available sequencing data obtained in Europe, Americas, Asia, and Africa: bovine* T. theileri*, ovine* T. melophagium*, and deer* T. cervi*. In general, the trypanosome species determination is not easy for the lack of discriminating morphological characters. In the past, many species have been defined principally on the basis of their epidemiological features, such as host and vector source, while nowadays modern biomolecular methods can help in the species delimitation but are not always conclusive [2]. The current status of the three above-mentioned trypanosome species constituting the* T. theileri* group is based mainly on their life cycle and host specificity, rather than on available sequencing data. Phylogenetic analyses support the existence of several genotypes and two main lineages (clades TthI and TthII), and some separation of the bovine, ovine, and cervid trypanosomes is accepted [3–6]; however, the clear separation and differentiation of these species have only a very weak support.

*Trypanosoma theileri* has been first identified in cattle in 1902 by Laveran and Bruce [1]. It is primarily found in cattle (*Bos taurus*), but other bovids such as bison (*Bison bison*) and water buffalo (*Bubalus bubalis*) [6] may be infected as well. In 1975, Kingston and Morton differentiated the trypanosomes found in a wapiti (*Cervus elaphus canadensis*; Wyoming, USA) from the cattle parasite* T. theileri* Laveran 1902 and named the new species* Trypanosoma cervi* Kingston and Morton, 1975 [7]. Since that time,* T. cervi* has been identified worldwide in mule deer (*Odocoileus hemionus*,* O. virginianus*), elk (*Alces alces*), reindeer (*Rangifer tarandus*), red deer (*Cervus elaphus*), fallow deer (*Dama dama*), and roe deer (*Capreolus capreolus*). Previous records of* T. theileri* and* T. theileri*-like infections based only on parasite morphology in various European wild deer populations may, in fact, represent finding of morphologically hardly differentiable* Trypanosoma cervi*. The last member of the* T. theileri* trinity,* Trypanosoma melophagium* described by Flu in 1908, is strictly restricted by its host specificity on sheep. Trypanosomes of the subgenus* Megatrypanum* are hardly identified in vertebrate host blood, and even isolation in culture is not an easy tool for their identification. In the case of* T. melophagium* it is peculiar that the trypanosomes were easy to find in huge numbers in sheep keds, the insect host, whereas infection in sheep, the mammal host, is rather cryptic.

Trypanosomes are primarily dixenous parasites principally transmitted by insects (from orders Diptera, Heteroptera, or Siphonaptera) and leeches. Members of the* T. theileri* group circulate between wild or domestic ruminants and their invertebrate vectors. While* T. theileri* is transmitted by tabanid flies, sheep keds are the only known vectors for* T. melophagium*. The situation in* T. cervi* is more complicated and the existence of several trypanosome species among different cervid hosts and/or different species in the same hosts from distant regions cannot be ruled out. It is assumed that the principal vector is the deer ked (*Lipotena* spp.), but tabanid flies are also mentioned as possible vectors; however, the existence of other vectors is also considered [6]. Only one report suggests that tabanids are also potential vectors due to cross infection among cattle and cervids using the gut content of tabanid flies. Regarding all morphological and poor molecular data from cervid trypanosomes, it seems clear that the evolutionary history of this trypanosomes is very complex and far from being elucidated.

Trypanosome parasites have not been reported from Italian sand flies. In 2016, screening for* Leishmania* parasites in sand flies collected in Bologna province, we isolated an unknown trypanosomatid, which was named TrPhp1 (Trypanosoma-Phlebotomus perfiliewi-1). The aim of this study was to characterize this trypanosomatid, isolated from a sand fly female of* Phlebotomus *(*Larroussius*)* perfiliewi* Parrot, 1930.

## 2. Methods

### 2.1. Collection of Sand Flies and Isolation of Flagellates

**Figure 1:**
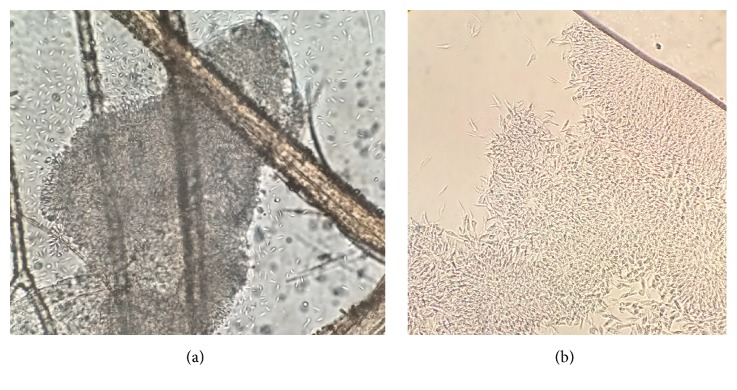
Photographs of the isolated trypanosome strain (TrPhp1) as was originally found in the sand fly hindgut (a) and in culture (b); light microscope (400×).

In the frame of leishmaniasis surveillance, sand flies were collected in a site located in the Bologna province, Emilia-Romagna region, Italy. Collection was made on the 27-28^th^ of July 2016, with an attractive trap baited with carbon dioxide. From a total of 2,728 sand flies trapped, a subsample of 82 males was morphologically identified at optical microscope [8, 9] after clarification with chlorolactophenol, recording 79* Ph. perfiliewi* and 3* Ph. perniciosus*. A number of 128 females were anesthetized with cold, dissected in cold sterile saline, and the guts were observed microscopically (× 400 magnification). The hindgut of one specimen was heavily infected with a large agglomerate of flagellate protozoan, with characteristics of the family Trypanosomatidae (Figure 1(a), video (available here)). No blood remnants were observed in the gut. The sand fly specimen was identified morphologically as* Ph. perfiliewi* (Figure S1); this identification was confirmed by the sequence of the mitochondrial gene cytochrome* c* oxidase I (COI) obtained according to Hebert et al. [10] utilizing LCO1490 and HCO2198 primer pair (sequence deposited in GenBank as KY646194).

The detected flagellates were inoculated into Evans' modified Tobie's medium (EMTM) containing 15% defibrinated rabbit blood and later transferred to Schneider's medium supplemented with 10% fetal calf serum for mass cultivation and further characterization.

### 2.2. Morphological Characterization of the Isolate

**Figure 2:**
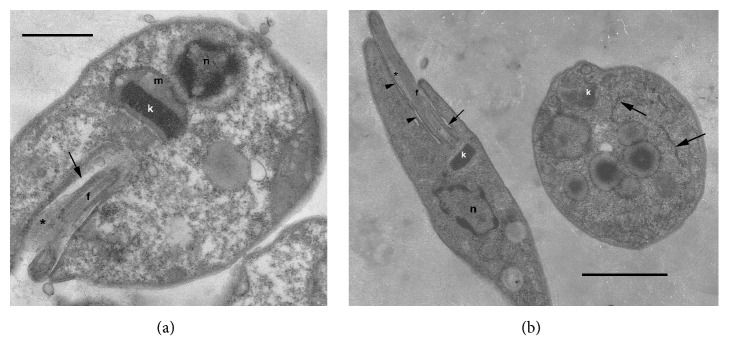
TEM micrographs of the culture stages of the isolated trypanosome strain (TrPhp1); epimastigotes (a, bb-left) and an amastigote (b-right). Arrow: flagellar pocket; white arrows: endoplasmic reticulum; arrow heads point to the area of adhesion of the flagellum to the cell body; asterisk: paraflagellar rod; f: flagellum; k: kinetoplast; n: nucleus; m: mitochondrion; bar: 1.65 *μ*m (a), 0.9 *μ*m (b).

Reared protozoa were firstly observed at light microscopy (400× magnification). To examine their ultrastructural features, aliquots of flagellate cultures were concentrated by centrifugation and prefixed in Karnovsky's fixative in cacodylate buffer (pH 7.2). After postfixation in 2% OsO_4_ in cacodylate buffer for 1.5 hours at 4°C, samples were washed in corresponding buffer, dehydrated in ethanol series, transferred to propylene oxide, and embedded in Epon 812. Semithin sections were stained with 0.05% toluidine blue in 1% sodium tetraborate and examined by optical microscopy. Thin sections (80nm) were stained with saturated uranyl acetate, followed by Reynolds lead citrate, and examined with Zeiss EM 900 electron microscope at 80 kV (Figure 2).

### 2.3. Biomolecular Identification and Phylogeny of the Isolate

Two markers were utilized to identify the TrPhp1 trypanosome, i.e., the ribosomal DNA internal transcribed spacer sequence (ITS-1) region of 320 bp and the 18S small subunit ribosomal RNA (SSU rRNA) gene of 927 bp. From the replicating parasite, DNA was extracted according to Rugna et al. [11], and then the two targets were amplified with the primer pair LITSR / L5.8S [12] for ITS-1 and the primer pair TRY927F / TRY927R [13] for the SSU rRNA, according to reported references. The obtained amplicons were purified with the AMPure XP PCR purification kit (Agencourt) and sequenced by CEQ 8000 sequencer (Beckman Coulter), using the GenomeLab DTCS Quick Start Kit (Beckman Coulter). The results were analyzed and assembled using CEQ 8000 version 8.0 software.

Homologue ITS-1 and SSU rRNA sequences of other trypanosomes were retrieved from GenBank with a BLAST search (https://blast.ncbi.nlm.nih.gov/Blast.cgi), aligned to the obtained sequences with the ClustalW algorithm, and then refined manually. Phylogenetic analysis was performed by software Beast 2 [14], which uses a Bayesian approach to infer phylogenetic tree. Between the available substitutions models, that with the lowest Bayesian information criterion score, estimated by MEGA6 [15], was selected (Tamura-Nei +G +I for SSU, Jukes-Cantor +G for ITS-1). The coalescent constant population was selected as priors with a chain length of 10 million. Obtained trees were summarized and shown by FigTree software. The same alignments were used to obtain trees with neighbor-joining and minimum evolution methods using MEGA6 [15].

## 3. Results

### 3.1. Morphological Characterization

A culture of trypanosomes was obtained from the gut of the specimen of* Ph. perfiliewi*. On slide smears prepared from the liquid culture, rosettes of flagellates were observed. The parasites showed a low degree of pleomorphism in culture, with abundant epimastigotes and scarce amastigotes. These trypanosomes differed from those observed in the slide directly obtained from the sand fly, which was smaller and rounded, with shorter flagella (Figure 1, Figure S2).

Transmission electron microscopy performed on cultured cells revealed the typical epimastigote morphotypes and rare amastigote forms (Figure 2). Epimastigotes were characterized by a centrally located nucleus, rod-shaped kinetoplast, and paraflagellar rod extending partially into the flagellar pocket.

### 3.2. Biomolecular Identification and Phylogeny of* Trypanosoma* sp.

The nucleotide sequences of the ITS-1 and SSU rRNA genes were submitted to GenBank and assigned the accession numbers KY672996 and KY681802, respectively.

BLAST searches of the sequences generated in this study suggested the parasite belongs to the genus* Trypanosoma*. The obtained ITS-1 sequence was of 223 bp and showed 100% similarity with three sequences belonging to the* Trypanosoma theileri*-like isolates D30 and TC2 (a.c.: AY773714, HQ664845, JN798601) from deer and sheep, respectively. The obtained SSU rRNA gene sequence of 819 bp showed 100% similarity with four sequences (a.c.: KJ195885, KJ195879, KJ397590 AJ009165), which were related to different* Trypanosoma theileri*-like isolates including D30, from* Cervus sp. *and* Dama dama*.

**Figure 3:**
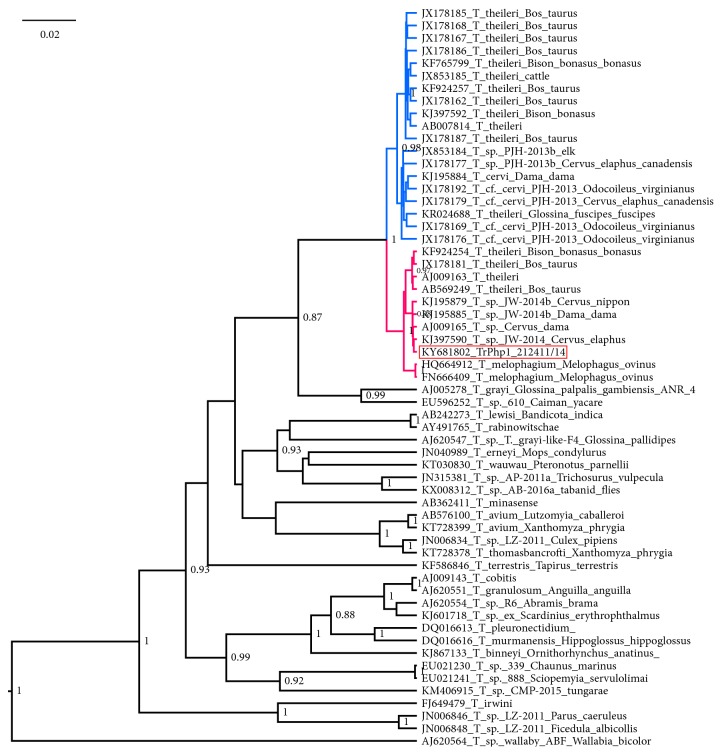
An SSU rRNA-based Bayesian phylogenetic tree demonstrating the position of newly obtained trypanosome (strain TrPhp1) of the* Trypanosoma theileri* group isolated from a sand fly female captured in Italy. For the other sequences in the tree the GB number is reported along with the* Trypanosoma* species and source of isolations, if available. Only posterior probabilities over 0.8 are shown at the node. Scale bar in units of substitutions per site. TthI clade in blue, TthII clade in red, font of sequence of isolated trypanosome with red background.

In the SSU rRNA tree (Figure 3), the* T. theileri* group is separated into two well-supported clades, previously named lineages TthI and TthII [5]; the sequence obtained in this study cluster is the lineage TthII along with sequences classified as* T. melophagium* and* T. theileri*. The only species consistent in the topology of this tree is* T. melophagium *(maximum posterior probability), while—as already suggested [2]—to define other systematic units, additional epidemiological data are needed, as host and vector specificity. This result confirms that SSU rRNA lacks sufficient resolution to discriminate among closely related species [16]. The obtained Bayesian tree is consistent with trees obtained by other methods employed (Figure S3).

**Figure 4:**
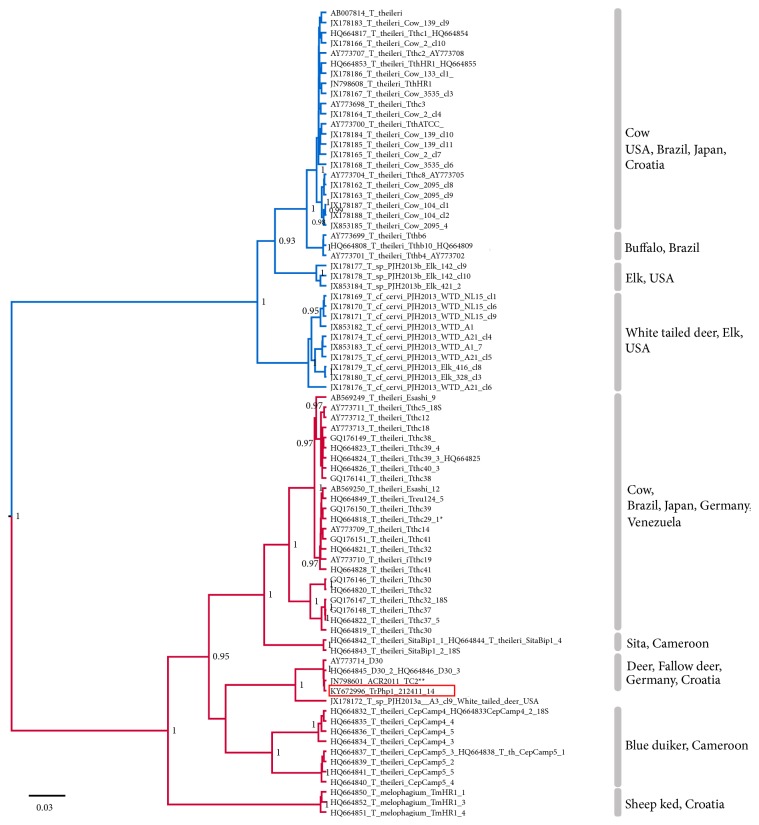
Phylogenetic tree of the ITS-1 sequence of isolated trypanosome (strain TrPhp1) and homologue sequences deposed in GenBank (GB numbers reported near the taxon name). Source and countries of isolation of the strains are reported near the square brackets. The tree was reconstructed using the Bayesian method. Only posterior probabilities over 0.8 are shown at the node. Scale bar in units of substitutions per site. TthI clade in blue, TthII clade in red, sequence of isolated trypanosome with red background. Identical sequences available in GenBank: *∗*HQ664829 TthcV2-7; HQ664847 Treu124-2; HQ664848 Treu124-3; *∗∗*JN798602 TC3; JN798603 TC4; JN798604 TC9; JN798605, TC10; JN798606, TC339; JN798607, TC603.

A more detailed view inside the* T. theileri* group is provided by the ITS-1 tree (Figure 4). This tree bears several clades with high posterior probability, which are quite consistent with the species reported, while sequences classified as* T. theileri* are spread in different clades. In the ITS1-based phylogenetic tree, our sequences are included in a clade (with the maximum posterior probability) containing other European sequences (from Germany and Croatia), not yet defined at species level but detected in various cervids. Also this obtained Bayesian tree is consistent with trees obtained by other methods (Figure S3).

## 4. Discussion

To the best of our knowledge, no reports of the* Trypanosoma* genus in sand flies have been published in Italy; thus this is the first report of the genus* Trypanosoma* in a naturally infected female* Phlebotomus perfiliewi* in the country. Despite the fact that the phylogenetic analysis based on SSU rRNA and ITS-1 did not allow us to unambiguously identify the studied parasites at species level, the trypanosome isolated from sand fly definitely belongs to the* Trypanosoma *(*Megatrypanum*)* theileri* group and thus represents the first report of this trypanosome species in sand flies in general.

This group comprises trypanosomes of ruminants with bovine* T. theileri* assumed as a type-species [17]. As suggested by Votýpka et al. (2015), without additional information about host specificity, the isolated* Trypanosoma* should be considered an operational taxonomic unit (OTU) within the* T. theileri* group. Phylogenies inferred in this study placed the newly obtained isolate TrPhp1 in the* T. theileri* lineage II, which comprised* T. melophagium* and trypanosome genotypes detected in cervids and cattle [6]. Interestingly, analysis of ITS-1 showed high sequence homology with trypanosomes detected in fallow deer (*Dama dama*) and red deer (*Cervus elaphus*) in Germany and Croatia, respectively [5, 17]. According to Garcia et al. [18], genotypes within the* T. theileri* group appear to be host specific. Based on the checklist of the Monteveglio Abbey Regional Park (Bologna province) and of the adjacent area, the only cervid species present at the sampled site is the European roe deer (*Capreolus capreolus*) and this information is consistent with the previous findings of* Megatrypanum* trypanosomes in this host species [19].

In the present study, the trypanosome of the* T. theileri* group was isolated from* Ph. perfiliewi*, a sand fly species broadly distributed in the Emilia-Romagna region, northeastern Italy [11, 20]. Natural infection of sand flies by trypanosomes was already reported; for example, trypanosomes of lizards and amphibians were detected in sand flies in Brazil [21, 22] and Ghana [23] and isolated from this insect in California [24] and Pakistan [25], incriminating phlebotomine sand flies as vectors of these protozoa. In addition, trypanosomes of birds and rodents were detected in sand flies in Peru [26] and Thailand [27], respectively, and trypanosomes of marsupials were isolated from sand flies in Brazil [28]. However, the commonly reported vectors of species belonging to the* T. theileri* group are tabanids and sheep or deer keds, and parasites are transmitted to vertebrate hosts via ingestion of vectors or via defecation. Therefore, the isolation of this trypanosome from a sand fly was unexpected. This finding could be explained by an underestimation of the vectorial competence of sand flies for* T. theileri* group trypanosomes and in line with the claim that also other vectors than tabanids and keds must be involved in the transmission of the* T. theileri* group [6]. Indirect support for the possible involvement of sand flies in the* T. theileri* group transmission is a massive occurrence of parasites in sand fly female gut after the defecation and the parasite localization in the posterior part of the digestive system, suggesting the possible transmission by defecation, the regular way of transmission occurring in the* T. theileri* group. Moreover, morphological forms present in the sand fly and in culture are consistent with the forms described in cycle of trypanosomes of this group, which pass from epimastigote to metacyclic stages in arthropods vectors [29, 30]. In such a case, the role of sand flies in the trypanosome transmission must be confirmed with an experimental infection of flies and transmission of parasites to vertebrate hosts. However, it is also possible that we recorded only a nonspecific development of trypanosome in the* Phlebotomus perfiliewi* sand fly female, which may represent a dead-end street for the parasite. This explanation could be supported, for example, by the regular occurrence of the* T. theileri* group in mosquitoes collected in several localities in Austria [31].

Although many trypanosome species have been known for years, complete life cycles and effect on vertebrate hosts have been elucidated only in a minority of the species. Insufficient knowledge of vectors and possible pathogenicity also concern trypanosomes from the* T. theileri* group.* T. theileri* belongs to a group of trypanosomes which are commonly considered nonpathogenic and may persist in cattle for many years without any evidence of clinical disease [32]. On the other hand, disease associated with* T. theileri* in cattle was sporadically reported [32, 33]. Even though the* T. theileri* group infections in roe deer and European elk and* T. theileri*-like infections in deer and chevrotains are documented, little is known about wild ruminants harboring these trypanosomes and being reservoirs for infection of livestock. For these reasons, the potential pathogenicity of the isolated trypanosome, its vertebrate host specificity, and development in possible vectors needs more investigation.

## 5. Conclusions

In this work we reported the unusual isolation of a trypanosome (the strain named TrPhp1), related to the* Trypanosoma theileri* group, from the sand fly* Phlebotomus perfiliewi*. Interestingly, in the dissected sand fly female the developmental stages of the parasite resembled the infective forms characteristic of this trypanosome group. Phylogenetic analysis highlighted a close relation between TrPhp1 and trypanosomes isolated from deer and fallow deer in Europe. These findings could indicate that life cycle of this trypanosome may involve sand flies as vectors and cervids as vertebrate hosts.

## Data Availability

The nucleotide sequences of the isolated trypanosome TrPhp1 were submitted to GenBank: ITS-1 with the accession number KY672996, SSU rRNA with the accession number and KY681802.

## Supplementary Materials

Supplementary Materials
** Figure S1.** The spermatheca of the* Phlebotomus perfiliewi* female from which the trypanosome strain (TrPhp1) was isolated; tip of the abdomen (above) and detail of the spermatheca (below).** Figure S2. **Cultured trypanosomes (strain named TrPhp1) stained (Hemacolor®) under optical microscope (1000×).** Figure S3.** Phylogenetic trees of the SSU and ITS-1 sequence alignments obtained by neighbor-joining and minimum evolution methods.** Video.** Video of the isolated trypanosome strain (TrPhp1) as was originally found in the sand fly hindgut.
