# Supplementary material for: Isolation of a Trypanosome Related to Trypanosoma theileri (Kinetoplastea: Trypanosomatidae) from Phlebotomus perfiliewi (Diptera: Psychodidae)
Source: Biomed Res Int. 2018 Jul 15;2018:2597074. doi: 10.1155/2018/2597074 (PMC6077689; doi:10.1155/2018/2597074)
Supplement: Supplementary Materials — Figure S1. The spermatheca of the Phlebotomus perfiliewi female from which the trypanosome strain (TrPhp1) was isolated; tip of the abdomen (above) and detail of the spermatheca (below). Figure S2. Cultured trypanosomes (strain named TrPhp1) stained (Hemacolor®) under optical microscope (1000×). Figure S3. Phylogenetic trees of the SSU and ITS-1 sequence alignments obtained by neighbor-joining and minimum evolution methods. Video. Video of the isolated trypanosome strain (TrPhp1) as was originally found in the sand fly hindgut. [file 2597074.f1.zip › mat.2597074.v2.pdf]

Supplementary Figures

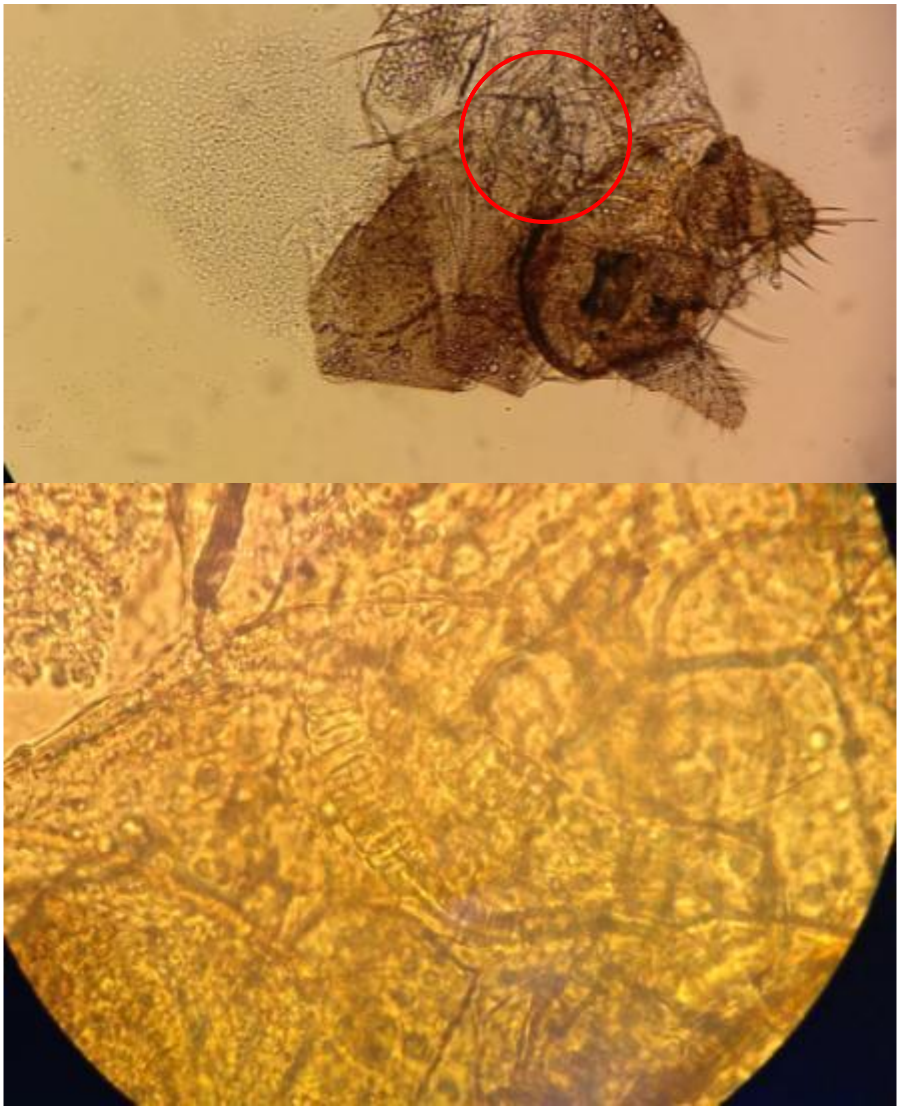

Figure S1. Figure S1. The spermatheca of the *Phlebotomus perfiliewi* female from which the trypanosome strain (TrPhp1) was isolated; tip of the abdomen (above) and detail of the spermatheca (below).

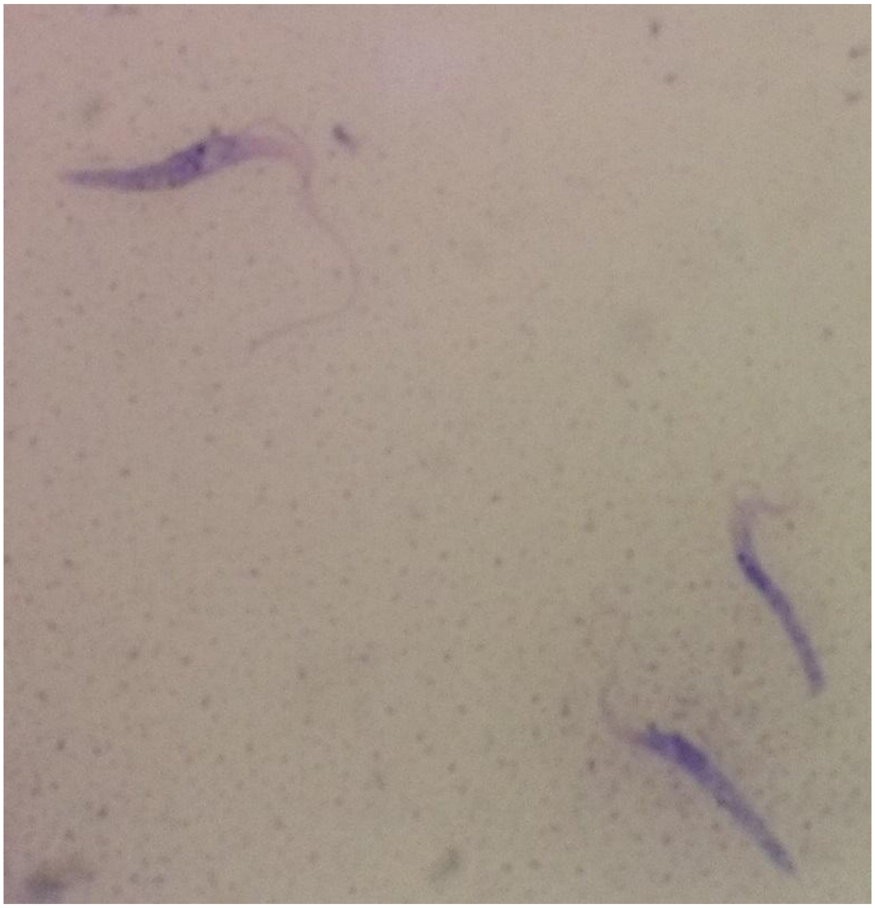

Figure S2. Cultured trypanosomes (strain named TrPhp1) stained (Hemacolor®) under optical microscope (1000×).

Figure S3. Phylogenetic trees of the SSU (a, b) and ITS-1 (c, d) sequence alignments obtained by neighbor-joining and minimum evolution methods. (a) Neighbor-Joining (LogDet evolutionary distance) and (b) Minimum Evolution (Kimura 2-parameter + G evolutionary distance) methods method. (c) Neighbor-Joining (LogDet evolutionary distance) and (d) Minimum Evolution (Kimura 2-parameter + G evolutionary distance) methods method. The percentage of replicate trees in which the associated taxa clustered together in the bootstrap test (1000 replicates) are shown next to the branches.

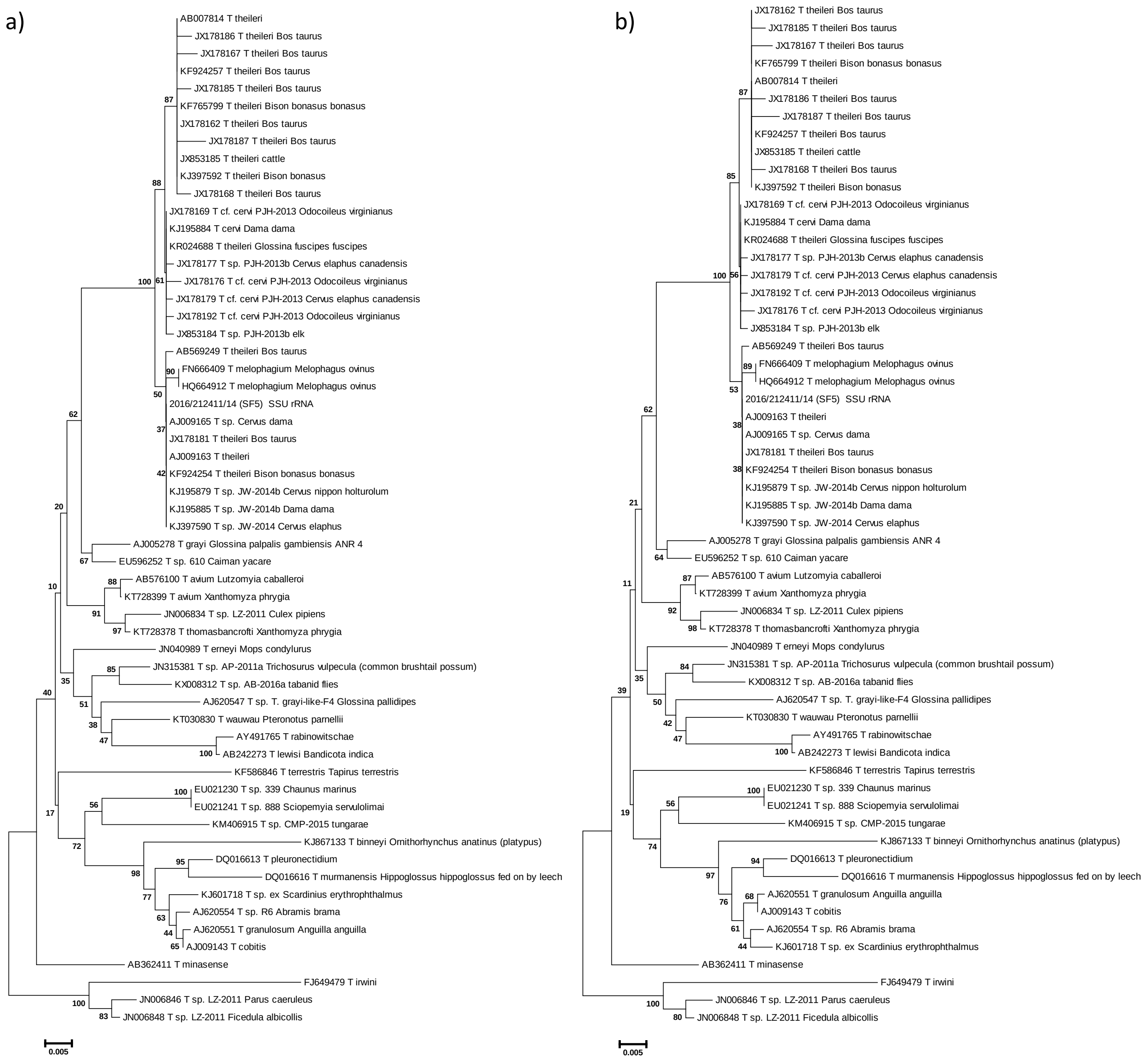

c)

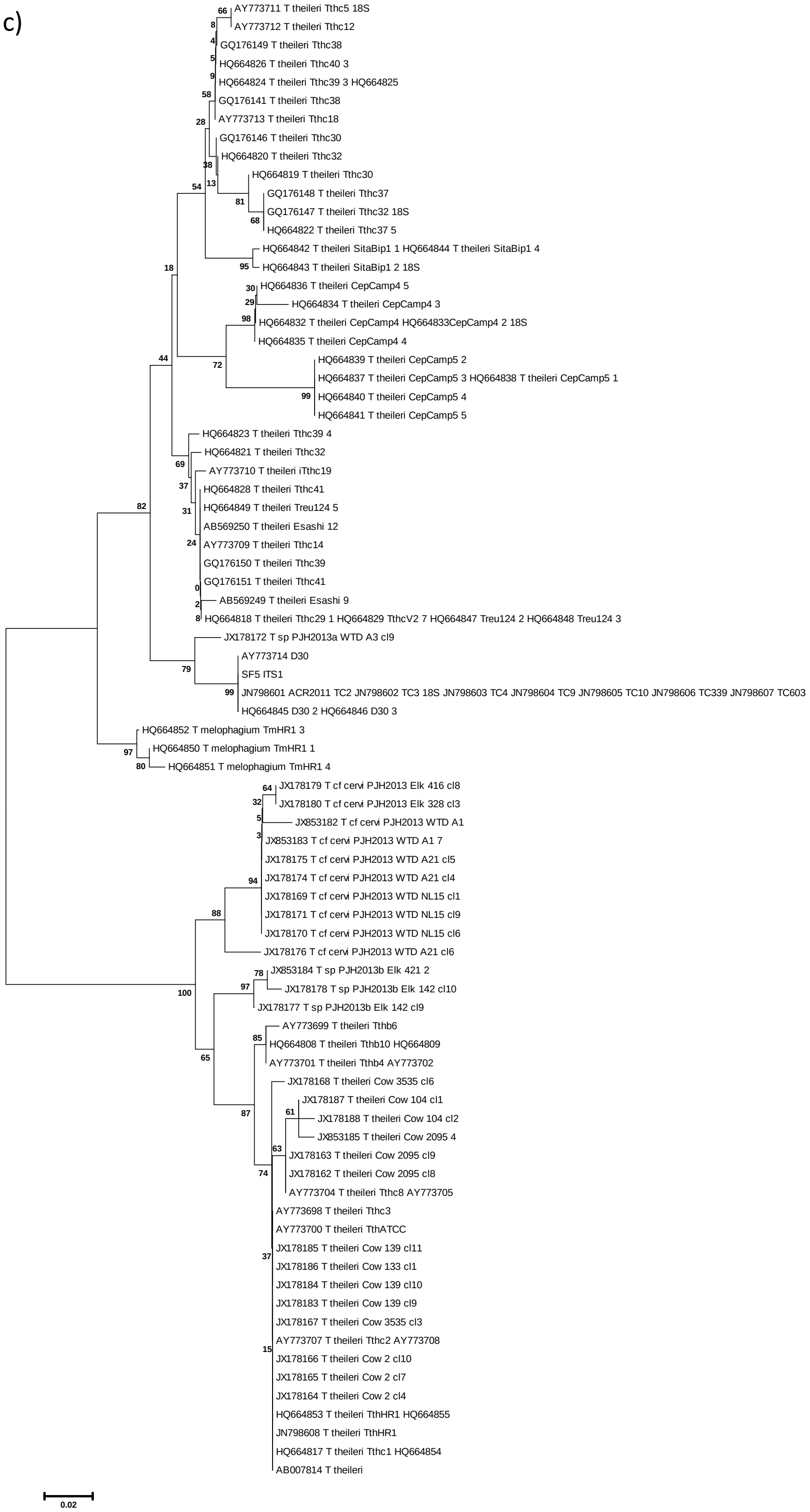
AY773711 T theileri Tthc5 18S
AY773712 T theileri Tthc12
GQ176149 T theileri Tthc38
HQ664826 T theileri Tthc40 3
HQ664824 T theileri Tthc39 3 HQ664825
GQ176141 T theileri Tthc38
AY773713 T theileri Tthc18
GQ176146 T theileri Tthc30
HQ664820 T theileri Tthc32
HQ664819 T theileri Tthc30
GQ176148 T theileri Tthc37
GQ176147 T theileri Tthc32 18S
HQ664822 T theileri Tthc37 5
HQ664842 T theileri SitaBip1 1 HQ664844 T theileri SitaBip1 4
HQ664843 T theileri SitaBip1 2 18S
HQ664836 T theileri CepCamp4 5
HQ664834 T theileri CepCamp4 3
HQ664832 T theileri CepCamp4 HQ664833CepCamp4 2 18S
HQ664835 T theileri CepCamp4 4
HQ664839 T theileri CepCamp5 2
HQ664837 T theileri CepCamp5 3 HQ664838 T theileri CepCamp5 1
HQ664840 T theileri CepCamp5 4
HQ664841 T theileri CepCamp5 5
HQ664823 T theileri Tthc39 4
HQ664821 T theileri Tthc32
AY773710 T theileri iTthc19
HQ664828 T theileri Tthc41
HQ664849 T theileri Treu124 5
AB569250 T theileri Esashi 12
AY773709 T theileri Tthc14
GQ176150 T theileri Tthc39
GQ176151 T theileri Tthc41
AB569249 T theileri Esashi 9
HQ664818 T theileri Tthc29 1 HQ664829 TthcV2 7 HQ664847 Treu124 2 HQ664848 Treu124 3
JX178172 T sp PJH2013a WTD A3 cl9
AY773714 D30
SF5 ITS1
JN798601 ACR2011 TC2 JN798602 TC3 18S JN798603 TC4 JN798604 TC9 JN798605 TC10 JN798606 TC339 JN798607 TC603
HQ664845 D30 2 HQ664846 D30 3
HQ664852 T melophagium TmHR1 3
HQ664850 T melophagium TmHR1 1
HQ664851 T melophagium TmHR1 4
JX178179 T cf cervi PJH2013 Elk 416 cl8
JX178180 T cf cervi PJH2013 Elk 328 cl3
JX853182 T cf cervi PJH2013 WTD A1
JX853183 T cf cervi PJH2013 WTD A1 7
JX178175 T cf cervi PJH2013 WTD A21 cl5
JX178174 T cf cervi PJH2013 WTD A21 cl4
JX178169 T cf cervi PJH2013 WTD NL15 cl1
JX178171 T cf cervi PJH2013 WTD NL15 cl9
JX178170 T cf cervi PJH2013 WTD NL15 cl6
JX178176 T cf cervi PJH2013 WTD A21 cl6
JX853184 T sp PJH2013b Elk 421 2
JX178178 T sp PJH2013b Elk 142 cl10
JX178177 T sp PJH2013b Elk 142 cl9
AY773699 T theileri Tthb6
HQ664808 T theileri Tthb10 HQ664809
AY773701 T theileri Tthb4 AY773702
JX178168 T theileri Cow 3535 cl6
JX178187 T theileri Cow 104 cl1
JX178188 T theileri Cow 104 cl2
JX853185 T theileri Cow 2095 4
JX178163 T theileri Cow 2095 cl9
JX178162 T theileri Cow 2095 cl8
AY773704 T theileri Tthc8 AY773705
AY773698 T theileri Tthc3
AY773700 T theileri TthATCC
JX178185 T theileri Cow 139 cl11
JX178186 T theileri Cow 133 cl1
JX178184 T theileri Cow 139 cl10
JX178183 T theileri Cow 139 cl9
JX178167 T theileri Cow 3535 cl3
AY773707 T theileri Tthc2 AY773708
JX178166 T theileri Cow 2 cl10
JX178165 T theileri Cow 2 cl7
JX178164 T theileri Cow 2 cl4
HQ664853 T theileri TthHR1 HQ664855
JN798608 T theileri TthHR1
HQ664817 T theileri Tthc1 HQ664854
AB007814 T theileri
0.02

d)

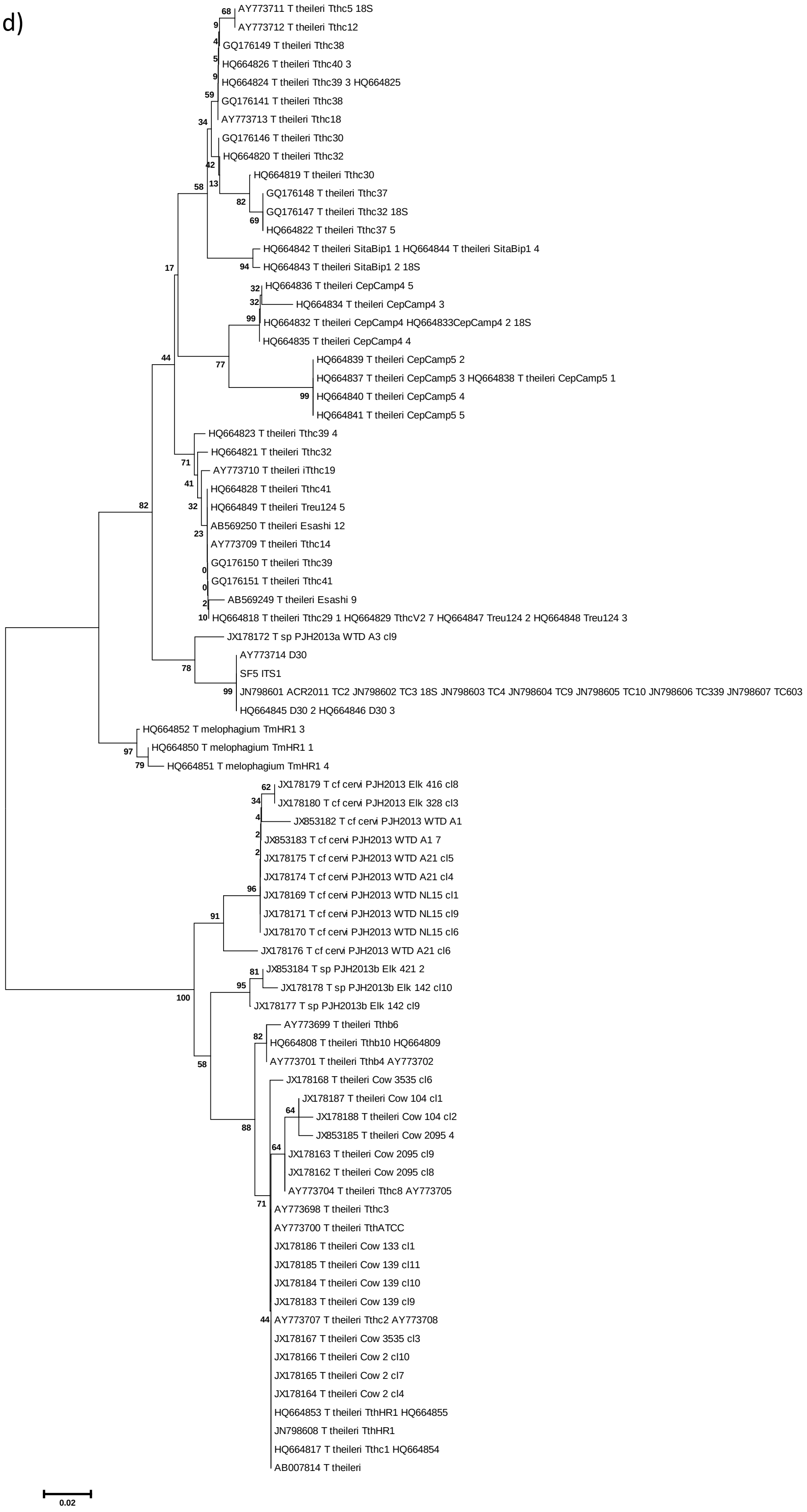
AY773711 T theileri Tthc5 18S
AY773712 T theileri Tthc12
GQ176149 T theileri Tthc38
HQ664826 T theileri Tthc40 3
HQ664824 T theileri Tthc39 3 HQ664825
GQ176141 T theileri Tthc38
AY773713 T theileri Tthc18
GQ176146 T theileri Tthc30
HQ664820 T theileri Tthc32
HQ664819 T theileri Tthc30
GQ176148 T theileri Tthc37
GQ176147 T theileri Tthc32 18S
HQ664822 T theileri Tthc37 5
HQ664842 T theileri SitaBip1 1 HQ664844 T theileri SitaBip1 4
HQ664843 T theileri SitaBip1 2 18S
HQ664836 T theileri CepCamp4 5
HQ664834 T theileri CepCamp4 3
HQ664832 T theileri CepCamp4 HQ664833CepCamp4 2 18S
HQ664835 T theileri CepCamp4 4
HQ664839 T theileri CepCamp5 2
HQ664837 T theileri CepCamp5 3 HQ664838 T theileri CepCamp5 1
HQ664840 T theileri CepCamp5 4
HQ664841 T theileri CepCamp5 5
HQ664823 T theileri Tthc39 4
HQ664821 T theileri Tthc32
AY773710 T theileri iTthc19
HQ664828 T theileri Tthc41
HQ664849 T theileri Treu124 5
AB569250 T theileri Esashi 12
AY773709 T theileri Tthc14
GQ176150 T theileri Tthc39
GQ176151 T theileri Tthc41
AB569249 T theileri Esashi 9
HQ664818 T theileri Tthc29 1 HQ664829 TthcV2 7 HQ664847 Treu124 2 HQ664848 Treu124 3
JX178172 T sp PJH2013a WTD A3 cl9
AY773714 D30
SF5 ITS1
JN798601 ACR2011 TC2 JN798602 TC3 18S JN798603 TC4 JN798604 TC9 JN798605 TC10 JN798606 TC339 JN798607 TC603
HQ664845 D30 2 HQ664846 D30 3
HQ664852 T melophagium TmHR1 3
HQ664850 T melophagium TmHR1 1
HQ664851 T melophagium TmHR1 4
JX178179 T cf cervi PJH2013 Elk 416 cl8
JX178180 T cf cervi PJH2013 Elk 328 cl3
JX853182 T cf cervi PJH2013 WTD A1
JX853183 T cf cervi PJH2013 WTD A1 7
JX178175 T cf cervi PJH2013 WTD A21 cl5
JX178174 T cf cervi PJH2013 WTD A21 cl4
JX178169 T cf cervi PJH2013 WTD NL15 cl1
JX178171 T cf cervi PJH2013 WTD NL15 cl9
JX178170 T cf cervi PJH2013 WTD NL15 cl6
JX178176 T cf cervi PJH2013 WTD A21 cl6
JX853184 T sp PJH2013b Elk 421 2
JX178178 T sp PJH2013b Elk 142 cl10
JX178177 T sp PJH2013b Elk 142 cl9
AY773699 T theileri Tthb6
HQ664808 T theileri Tthb10 HQ664809
AY773701 T theileri Tthb4 AY773702
JX178168 T theileri Cow 3535 cl6
JX178187 T theileri Cow 104 cl1
JX178188 T theileri Cow 104 cl2
JX853185 T theileri Cow 2095 4
JX178163 T theileri Cow 2095 cl9
JX178162 T theileri Cow 2095 cl8
AY773704 T theileri Tthc8 AY773705
AY773698 T theileri Tthc3
AY773700 T theileri TthATCC
JX178186 T theileri Cow 133 cl1
JX178185 T theileri Cow 139 cl11
JX178184 T theileri Cow 139 cl10
JX178183 T theileri Cow 139 cl9
AY773707 T theileri Tthc2 AY773708
JX178167 T theileri Cow 3535 cl3
JX178166 T theileri Cow 2 cl10
JX178165 T theileri Cow 2 cl7
JX178164 T theileri Cow 2 cl4
HQ664853 T theileri TthHR1 HQ664855
JN798608 T theileri TthHR1
HQ664817 T theileri Tthc1 HQ664854
AB007814 T theileri
0.02
